# Fabrication of Colored Polymeric Membrane Using Clay-Based Nano Pigments of Safranin O (SO) Dye

**DOI:** 10.3390/membranes13070619

**Published:** 2023-06-24

**Authors:** Chandra Mohan, Priyanka Kumari, Neeraj Kumari, Arvind Negi

**Affiliations:** 1 Department of Chemistry, School of Basic and Applied Sciences, K. R. Mangalam University Gurugram, Gurugram 122103, India; gurgaonmohan@yahoo.co.in; 2 Department of Chemistry, Shivaji College, University of Delhi, Delhi 110027, India; 3 Department of Bioproduct and Biosystems, Aalto University, 02150 Espoo, Finland

**Keywords:** nano pigments, clay, safranin O, polymeric membrane, adsorption

## Abstract

In the present work, a novel methodology was developed for the fabrication of clay-based nano pigments with enhanced thermal stability and used further as a colorant to prepare polymeric membranes. Initially, the batch extraction studies were performed to analyze the maximum adsorption of Safranin O (SO) dye onto pristine montmorillonite (Mt) and organo montmorillonite (OMt) by varying different parameters like pH, contact time, and concentration. It was confirmed from batch extraction studies that the adsorption efficacy of pristine Mt for SO was found to be more than OMt due to their negatively charged surface. Clay-based nano pigments were fabricated by considering the optimized condition where the maximum uptake of SO was observed and further characterized by XRD, FTIR, TGA, and SEM techniques. XRD studies confirmed the intercalation of SO dye while FTIR spectra revealed surface interaction of the dye with Mt/OMt. TGA studies showed that the clay-based nano pigments had more thermal stability than pure SO. Nano pigments were used as colorants to prepare thin, transparent, and homogeneously dispersed polymeric membranes through the solvent casting method. XRD studies of the polymeric membrane confirmed that the intercalation of poly methylmethacrylate (PMMA) into the interlayer of clay increases interlayer spacing, which was further confirmed by the TEM analysis. The mechanical properties of the PMMA polymeric membrane were also enhanced after the dispersion of clay-based nano pigments.

## 1. Introduction

Various types of clay and their derivatized dyes or pigments have been widely used as colorants to generate various colored design materials. Dyes and pigments are primary organic coating components [1,2]. The primary purpose of dyes and pigments is to color the coating materials and improvespecific operational properties. However, dyes and pigments also have some drawbacks. Most pigments show good colorimetric properties but suffer from poor dispersion of polymeric materials and low-grade heat and corrosion resistance. Organic dyes are easily dispersed in polymeric materials, but have poor durability, chromatic properties, and resistance against UV radiation [3,4,5,6]. Although the durability of synthetic colorants guarantees vibrant and long-lasting colors, the large amount of hazardous wastewater effluents presents a challenge for the sustainable production and processing of synthetic colorants (or dyes). Therefore, to overcome these limitations, clay-based nano pigments have been developed as innovative materials that integrate the excellent properties of dyes and pigments [7]. Clay-based nano pigments are hybrid materials that combine layered silicate particles and organic dyes. The clay particles immobilize the dye molecules minimizing the leaching of dyes (to the environemnt) and thereby improving the color strength [8]. Recent years have witnessed an upsurge in the usage of nano pigments offering applications such as coating, printing, and painting, and therefore become an alternative choice (as colorants) in the polymer industry owing to their ability to disperse (homogenously) into a polymer matrix to form thin colored polymer membranes [8,9]. Importantly studies also showed an improvement in reducing the ecotoxicity profile when such strategies are employed [10].

Hybrid nano pigments are primarilysynthesized using the ion exchange method, (generally occurs between cationic organic dyes and nano-clay) [11]. In the ion exchange method, cationic dyes interact with clay (particles) through chemical/electrostatic interaction and do not migrate from the polymeric matrix [12,13]. A rhodamine-B/montmorillonite nano pigment was synthesized by Raha et al. and confirmed the enhanced photo-stability of synthesized nano pigments compared to pure rhodamine-B dye [14]. The stability of a rhodamine6G intercalated montmorillonite nano pigment toward UV radiation and minerals, acids, and bases was investigated by Smith et al. (2011) and confirmed the enhanced stability of nano pigments [15]. Mahmoodi et al. synthesized high-performance blue nano pigments using methylene blue dye and Closite 15A, which was applied further as a coating material in the epoxy formulation [16]. A series of organic–inorganic lake pigments were prepared using natural carminic acid and alizarin organic dyes through immobilization onto Ti and Al-pillared montmorillonite minerals which were found to be more stable against light [17].

Another hybrid pigment of carminic acid with improved light stability was synthesized using saponite and montmorillonite clay. The synthesized hybrid nano pigment was covered with polyorganosilane, an additional protective layer against radiation [18].

The main objective of the present work was to synthesize clay-based nano pigments with enhanced thermal stability through the batch extraction method. Further, it used as a colorant to prepare a polymeric membrane. Initially, montmorillonite (Mt) clay and its modified organic forms (OMt) were used as adsorbents for the removal of non-biodegradable cationic dye (Safranin O (SO)) from aqueous media. The dye-adsorbed clay was further used to prepare the colored polymeric membranes. No research article has previously reported the synthesis of nano pigments through the batch extraction method, but research articles have notified the synthesis of clay-based nano pigments through the ion exchange method without showing the effect of pH, contact time, and concentration, which were further used as colorants to form a colored, transparent, and homogeneously dispersed polymeric film with enhanced mechanical properties.

Safranin O dye is classified as a cationic dye, and because of positive- charged aromatic nitrogen (phenazine ring), it usually decomposes chemically. Although Saranin O is one of the oldest dyes used in different industries for various purposes, it has a high ecotoxicity profile [19]. Various clinical studies found significant Safranin O dye-induced human toxicities. (neurotoxicity, nephrotoxicity, and hepatotoxicity). Safranin O dye affects the growth of aquatic life forms and reduces the efficacy of the photosynthesis process,. However techniques including (electrodialysis, reverse osmosis, nanofiltration, Fenton’s reagent, etc.) are known to remove Safranin O dye from water bodies, but those methods (that utilize adsorption in principle) are the common ones due to their efficiency and cost effectivity [20,21]. Therefore, researchers continuously seek to develop adsorbents, (activated carbon, and metal oxide nanoparticles) that are most efficient and cost effective in removing the dyes from water bodies. Halboos and Hussain used nano iron oxide (Nano FeO) to remove Safranin O dye from aqueous media [19]. Azimvand et al. (2018) synthesized lignin nanoparticle-g-polyacrylic acid in the presence of potassium persulfate as the radical initiator and used it as an adsorbent for the removal of Safranin O dye (138.88 mg g^−1^) [22]. Das et al. (2021) compared the adsorption efficiencies of coconut coir and its acid-treated forms (phosphoric acid and sulfuric acid) for the removal of Safranin O dye where they confirmed the maximum efficiency of phosphoric acid-treated coconut coir [23]. Parmar and Shah observed 97% of Safranin O dye adsorbed on the surface of tamarind seeds [24]. Didehban et al. (2016) compared the adsorption capacities of lignin nanoparticles and a polyacrylic acid hydrogel for the adsorption of Safranin O dye and confirmed a 1666.6 mg g^−1^ adsorption of dye onto the polyacrylic acid hydrogel [25]. 

Among adsorbents, clay is one of the most used adsorbents and at a large scale for removing pollutants from water bodies (due to its low cost, high adsorption efficiency, specific surface area, and availability). The central structural unit of clay minerals is the tetrahedral and octahedral units. Based on the arrangement of the tetrahedral and octahedral unit, clay minerals are categorized into three categories: 1:1 (one tetrahedral and one octahedral unit); 2:1 (one octahedral unit sandwiched between two tetrahedral units); 2:1:1 (2:1 clay layer present with tri-octahedral sheet). All of the clay minerals are negatively charged due to the isomorphous substitution of aluminum ions in the place of silicon ions in the tetrahedral sheet and magnesium ions in the place of aluminum ions in octahedral sheets [26]. Due to the negatively surface-charged clay minerals, they possess reasonable adsorbent properties for cationic dyes, compared to their adsorbent properties for other dyes. Li et al. (2020) used palygorskite, sepiolite, and clinoptilolite to remove cationic dyes and Safranin O [27]. The binary solution of Safranin O and Toluidine B dyes was used to investigate the adsorption efficiency of zeolite, where 96 mmol/kg and 126 mmol/kg of dye adsorption were observed [28].

## 2. Materials and Methods 

### 2.1. Material Used

All of the chemicals used during the experiment were of analytical reagent grade. Safranin O and montmorillonite clay were obtained from Sigma Aldrich, St. Louis, MO, USA. Cetylpyridinium chloride, a cationic surfactant, was purchased from Merck Pvt. Ltd., Mumbai, India. 

### 2.2. Synthesis of Organo Montmorillonite

**Figure 1:**
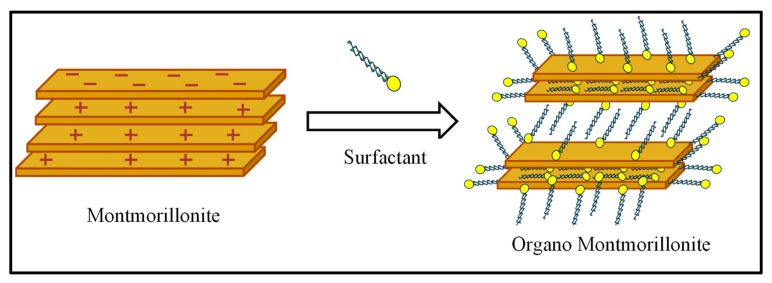
Possible interaction of a cationic surfactant with Mt.

The organoclay (OMt) was prepared through the ion exchange process by modifying a reported procedure [15]. As the clay was treated with the CPC surfactant, CPC could be intercalated in the interlayer of clay by replacing exchangeable ions and can form monolayer, bilayer, and paraffin complexes that can be further confirmed by XRD studies, depending upon the interlayer spacing (Figure 1) [29]. 

### 2.3. Preparation of Stock Solution of SO Dye

A standard stock solution of dye (1000 mg L^−1^) was prepared using double distilled water. The solutions of the required concentrations were prepared by diluting the stock solution and used for further experiments. All of the experiments were conducted at room temperature in a 25 mL dye solution of the required concentration using 0.1 g adsorbents (Mt and OMt). The uptake of dye adsorbed onto pristine and organo clays was estimated as:

(1) $$q_{e}=\frac{{(C}_{i}-C_{e})\times V}{m}$$

where *q_e_* (mg g^−1^) is the amount of SO adsorbed onto pristine and organo clays; *C_i_* (mg L^−1^) is the initial concentration of SO; *C_e_* (mg L^−1^) is the concentration of SO in solution at equilibrium; *V* (L) is the volume of the aqueous solution of SO; *m* (g) is the amount of the adsorbent. 

### 2.4. Synthesis of Clay-Based Nano Pigments 

As the Introduction section discusses, nano pigments are composite materials synthesized using clay and organic dyes. 

**Figure 2:**
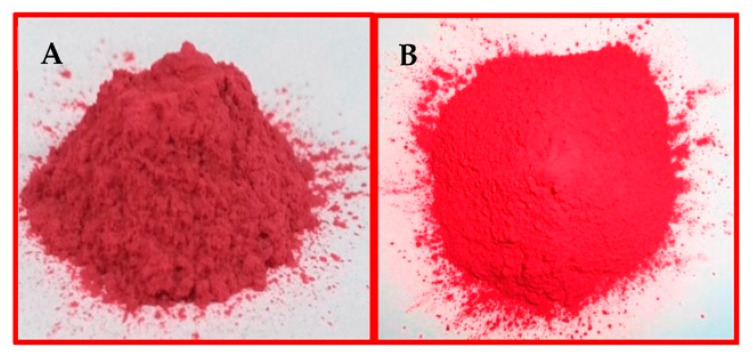
Clay-based nano pigments: (**A**) Mt-based nano pigments; (**B**) OMt-based nano pigments.

Clay-based nano pigment were synthesized by considering the optimized pH, contact time, and concentration obtained during batch extraction studies. To prepare clay-based nano pigments, 1 g of clay/organo clay was added to the dye solution after adjusting the pH and left to stir on the mechanical shaker for optimized time. In the last, centrifugation of the reaction mixture was carried out for 20 min at 8000 rpm to separate the residue from the supernatant. The resultant residues were dried at 80 °C overnight in an oven. The obtained residues were designated as clay-based pigments, as shown in Figure 2.

### 2.5. Characterization 

A mechanical shaker (Metalab Rotary Shaker, Uma Pharmatech Machinery, Ahmadabad, Gujarat, India) with speeds from 140 rpm to 300 rpm was used in the present study for the adsorption of dye. X-ray diffraction (XRD) was conducted on a Philips X′ Pert-PRO PMRD (model 3040160, California, USA) system using Cu Kα radiation (n = 1.54056 Ǻ) at 50 kV and 100 mA at a scanning speed of 0.008°/s in continuous scan mode operating at 2θ values between 2 and 40°. A Perkin-Elmer FTIR spectrophotometer (RX I (RX-1) model, American Laboratory Trading, Groton, CT 06340, USA) was used to record the FTIR spectra using the KBr matrix at room temperature over the wavenumber range 4000–400 cm^−1^, employing a total of 64 scans at a resolution of 4 cm^−1^. Thermal analysis was carried out using TGAQ-500, (Tga 8000, SIM GmbH, Laubach, Germany) in a nitrogen atmosphere, flow rate of 10 mL/minute, at the temperature of 30 °C–800 °C at a rate of 10 °C/minute. Surface images of the synthesized samples were recorded using the scanning electron microscopic technique. One drop of the aqueous dispersion of samples was mounted on stubs, air-dried, and sputter-coated with gold in a vacuum evaporator and photographed using a scanning electron microscope (ZEISS EVO 40, ZEIS S, Jena, Germany) coupled with an accelerating voltage of 30 KV. The transmission electron microscopy (TEM) image of the PMMA polymeric membrane was generated using a JOEL, Model-JEM-2100 (Japan) transmission electron micro analyzer with an accelerating voltage of 200 KV. Pure PMMA and a nano pigment-based PMMA polymeric membrane with different inorganic fillers were tested for their tensile strength, and the Young’s modulus (mechanical characteristics; dimensions: 50 mm × 15 mm × thickness: 0 mm to 23 mm) was recorded using a tensile testing machine (Instron UTM 3369). The tensile strength of the PMMA films was calculated using the following formula:

(2) $$Tensile\;strength=\frac{Force\;(maxiumum\;load\;applied\;to\;elongate\;the\;film)}{Area\;(width\times thickness)}$$

## 3. Results and Discussion

### 3.1. Effect of pH on SO Stability in Aqueous Media

**Figure 3:**
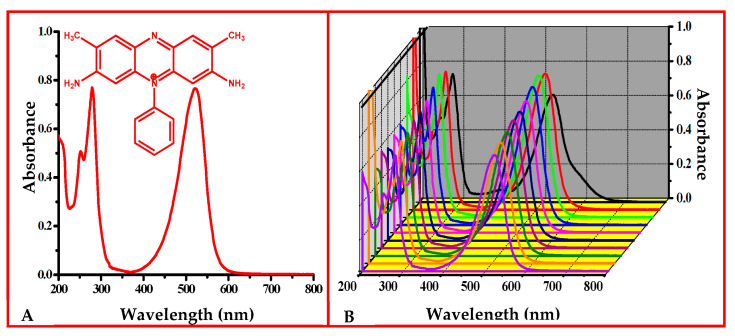
(**A**) UV–Vis spectrum of SO in an aqueous solution at its natural pH (5.2). (**B**) Effect of pH on the stability of the SO dye in an aqueous solution.

The electronic absorption spectrum of SO mainly showed three absorption bands at 251, 278, and 521 nm in aqueous media (Figure 3). The effect of pH on the electronic absorption spectrum of SO was examined. At pH 1, the λ_max_ value was slightly different (i.e., 525 nm), which might have been due to the protonation of all the nitrogen present in SO and from pH 2 to 10, the λ_max_ value remained the same (i.e., 521 nm). However, the absorbance value at the respective λ_max_ appeared to be influenced by changing the pH of the SO dye solution [30]. 

### 3.2. Batch Extraction Studies 

**Figure 4:**
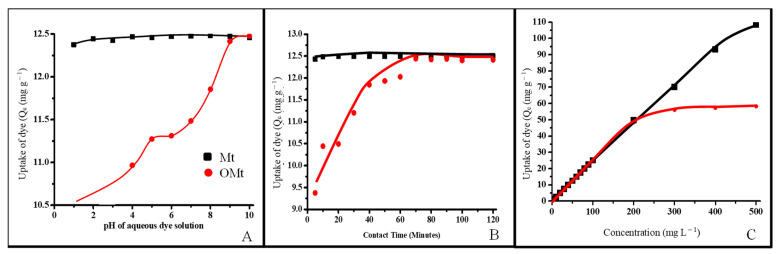
(**A**) Adsorption of SO as a function of pH. (**B**) Adsorption of SO as a function of contact time. (**C**) Adsorption of SO as a function of the initial concentration of dye solution.

The interaction of SO with pristine and organo Mt was studied as a function of pH of the aqueous solution of SO, contact time, and initial concentration of the aqueous solution of SO (Figure 4). 

#### 3.2.1. Adsorption of SO as a Function of pH

Figure 4A shows the effect of pH on the uptake of SO by Mt and OMt. The adsorption was conducted at different pH (pH 1–10) where the concentration of the dye solution (50 mg L^−1^) and time of shaking (30 min) was kept constant. The maximum adsorption of SO with Mt and OMt was found at pH 8 and 10 (12.5 mg g^−1^), respectively. As the pH of the aqueous solution of SO increased, the negative charge on the surface of Mt/OMt increased due to the presence of OH^−^ ions. Therefore, the maximum interaction of SO with Mt was found in the basic medium.

#### 3.2.2. Adsorption of SO as a Function of Contact Time

During adsorption of the SO dye as a function of contact time, the concentration of the dye solution was 50 mg L^−1^ and left to shake on a mechanical shaker by varying the time (5–120 min) after adjusting the pH (pH 8 in the case of Mt and pH 10 in the case of OMt). A total of 12.5 mg g^−1^ of SO was retained by Mt within 40 min whereas 12.3 mg g^−1^ of SO was retained by OMt within 70 min of contact time (Figure 4B). The uptake of SO was rapid during the initial stage of contact time and almost became constant after attaining the equilibrium stage, which might be due to the non-availability of adsorption active sites.

#### 3.2.3. Adsorption of SO as a Function of Initial Concentration

As the concentration of the aqueous solution of SO increased from 5 mg L^−1^ to 500 mg L^−1^, the uptake of SO increased from 1.24 mg g^−1^ to 108 mg g^−1^ (at 500 mg/L) in the case of Mt, and 1.23 mg g^−1^ to 58 mg g^−1^ (at 300 mg L^−1^) in the case of OMt. The maximum adsorption of SO was retained by pristine Mt compared to organo OMt due to their negatively charged surface, resulting in the electrostatic interaction between the positively charged SO and negatively charged Mt, which is not possible for OMt due to the CPC surfactant present on the surface of Mt. 

### 3.3. XRD Studies of Mt and OMt Based Nano Pigments

**Figure 5:**
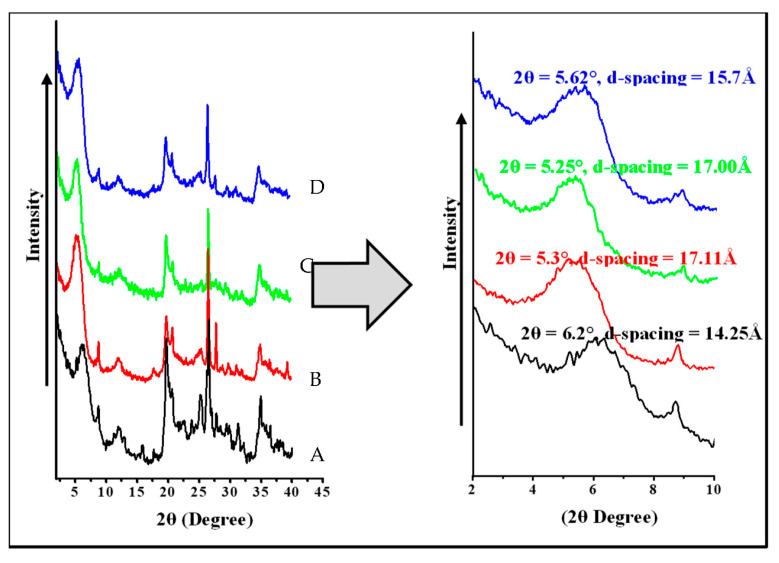
XRD pattern of (A) pristine Mt, (B) OMt, (C) Mt-based nano pigment, and (D) OMt-based nano pigment.

The XRD pattern of pristine Mt showed a distinct diffraction peak at 2θ = 6.2°, confirming 14.25 Å basal spacing (d-spacing) (Figure 5). The basal spacing increased (17.11 Å) after treatment with CPC as the diffraction peak of Mt shifted toward a lower angle (2θ = 5.3°), which further confirmed the intercalation of CPC in the interlayer of Mt.

The diffraction peak of Mt shifted toward a lower angle (2θ = 5.25°) after the adsorption of SO, indicating an increment of basal spacing of 17.00 Å, thus confirming the presence of SO in the interlayer of Mt.

However, in the case of organo Mt-based nano pigments, there was a shift of the 2θ angle toward the higher angle from 5.3° to 5.62°, meaning that there was a decrease in the d-spacing from 17.11 Å to 15.7 Å, which may be due to either the agglomeration of organo Mt in the dye solution or intercalation of SO in the interlayer of organo Mt by substituting the surfactant moiety. The dye adsorption on the surface was due to weak hydrogen bonding or van der Waals interactions between the dye molecule and O-plane of the outer surface of the clay layer [31,32].

### 3.4. FTIR Spectral Studies of Mt- and OMt-Based Nano Pigments 

**Figure 6:**
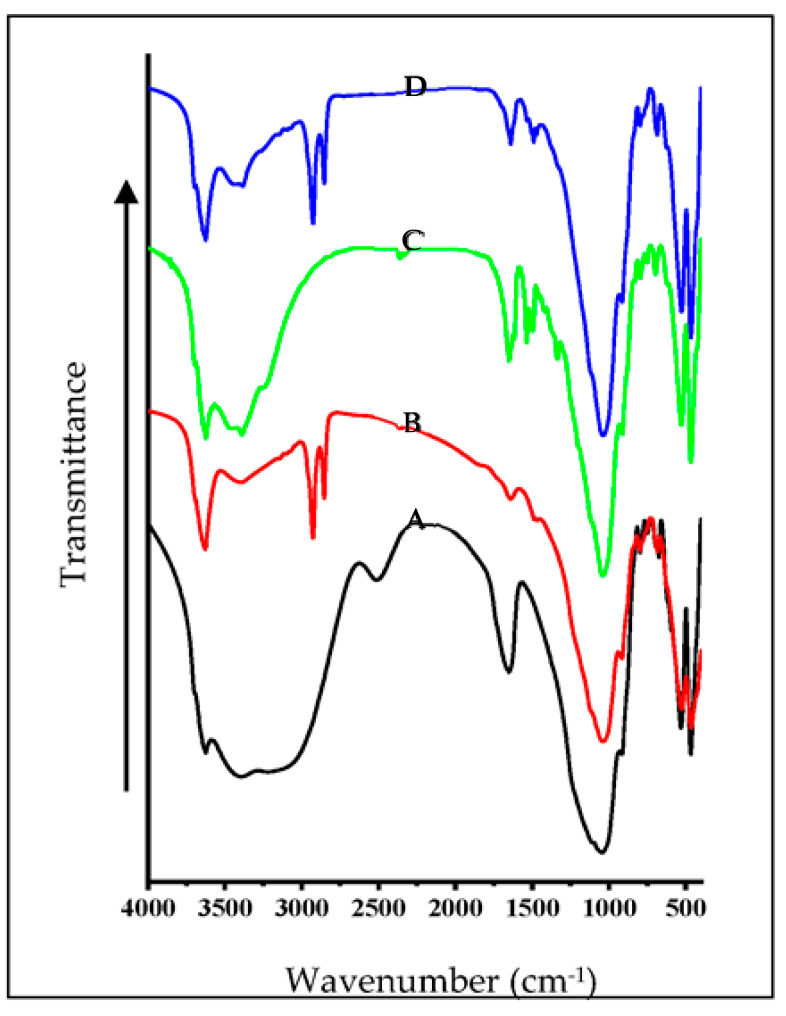
FTIR spectra of (A) pristine Mt, (B) OMt, (C) Mt-based nano pigment, and (D) OMt-based nano pigment.

The FTIR spectra of Mt and OMt before and after dye adsorption are shown in Figure 6. The FTIR spectrum of Mt showed an O–H stretching vibration band of the structural O–H group at 3624 cm^−1^ and the H–O–H stretching vibration band of the interlayer water in Mt at 3410 cm^−1^. The vibrational band at 1648 cm^−1^ was due to water’s H-O-H bending vibration on the surface or in the interlayer region. A broad band centered around 3083 cm^−1^ in Mt was due to the H–O–H stretching vibration surface adsorbed water of Mt and the band at 2510 cm^−1^ was due to surface and/or interlayer water arising because of the combination of two vibrational frequencies of water. The strong stretching vibrational band of Si–O–Si and Si–O–Al for Mt was observed at 1043 cm^−1^. 

On interaction with CPC, a new pair of vibrational bands at 2929 cm^−1^ and 2858 cm^−1^ was observed for Mt, which was due to the asymmetric and symmetric vibrations of the methylene group of the CPC, confirming the presence of CPC on the surface of OMt. The intensity of the H–O–H stretching vibrational band (at 3410 cm^−1^) was reduced substantially, confirming the loss of the interlayer water by CPC. Therefore, the FTIR spectra revealed that CPC was present on the surface as well as in the interlayer region of Mt.

In the case of Mt-based nano pigments, the vibrational bands at 3083 cm^−1^ and 2510 cm^−1^ disappeared, confirming the interaction of TFT with Mt. The vibration band around 1480 cm^−1^ confirmed the presence of the aromatic ring of SO. There was no substantial change in the vibration bands from 4000 cm^−1^ to 2000 cm^−1^ in the case of the OMt-based nano pigments, which may be due to the low uptake of SO onto Omt. In contrast, a pair of new vibration bands at 1485 cm^−1^ and 1472 cm^−1^ appeared due to C=C stretching vibration of the aromatic ring present in SO, resulting in the surface interaction of SO [33]. 

Therefore, based on the above result, it can be concluded that SO was found on the surface of the pristine clay and organo clay.

### 3.5. Thermogravimetric Analysis (TGA) of Mt- and OMt-Based Nano Pigments

**Figure 7:**
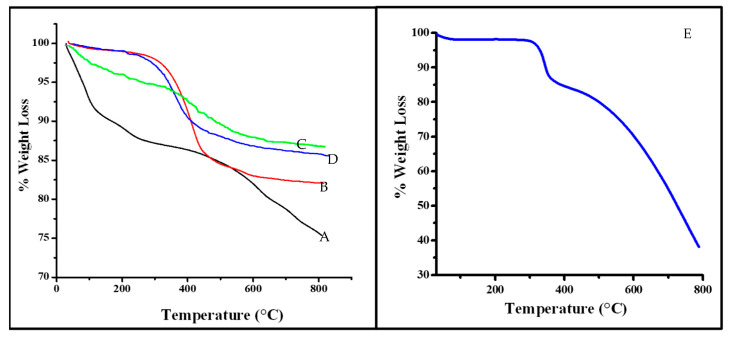
Thermogravimetric analysis of (A) pristine Mt, (B) OMt, (C) Mt-based nano pigment, (D) OMt-based nano pigment, and (E) pure SO dye.

Thermogravimetric analysis of the clay and organo clay-based nano pigments is shown in Figure 7.

In the case of pristine Mt, 8.8% weight loss was observed between 30 °C and 130 °C, due to the elimination of physically adsorbed and interlayer water. The second weight loss (4.91%) was due to the dehydroxylation observed between 130 °C and 400 °C. A total of 10.41% weight loss was observed over 400 °C, due to dehydroxylation of the structural –OH groups present in clay. 

The thermal analysis of Mt increased after treatment with CPC. In the case of OMt, the loss of physically adsorbed water decreased (1.61%) over the temperature range of 30 °C to 130 °C compared to the pristine Mt. However, the TG curve of OMt showed a lack of interlayer water and a substantial reduction in surface adsorbed water due to the presence of CPC, which further supports CPC intercalation into the clay interlayer and onto its surface. The weight loss due to the removal of organic species (i.e., surfactant molecule) was 12.77% over the temperature range of 130 °C−450 °C. A weight loss of 3.13% was observed between 450 °C and 800 °C due to the loss of the structural hydroxyl group within clay [34,35].

The TG curve of pure SO showed 3% weight loss between 30 °C and 200 °C, which was due to the physically adsorbed water. The drastic weight loss of around 65% observed above 200 °C showed that the decomposition of organic dye was due to the oxidation of carbon, hydrogen, and nitrogen in the form of H_2_O, CO_2_, and NO_2_. 

In the case of the Mt-based nano pigments, the TG curve showed only a 3.22% mass loss up to 200 °C due to dehydration, 2.4% weight loss in the temperature range of 200–500 °C from the decomposition of organic dye, and a further 3.84% weight loss possibly due to the dehydroxylation of clay. In the case of the organo Mt–SO dye, the weight loss decreased (0.94%) between 30 °C and 200 °C. There was a slight reduction in the thermal stability of OMt after interaction with SO, which might be due to SO decomposition present in the interlayer of OMt (as discussed in the XRD studies).

### 3.6. Morphological Studies

**Figure 8:**
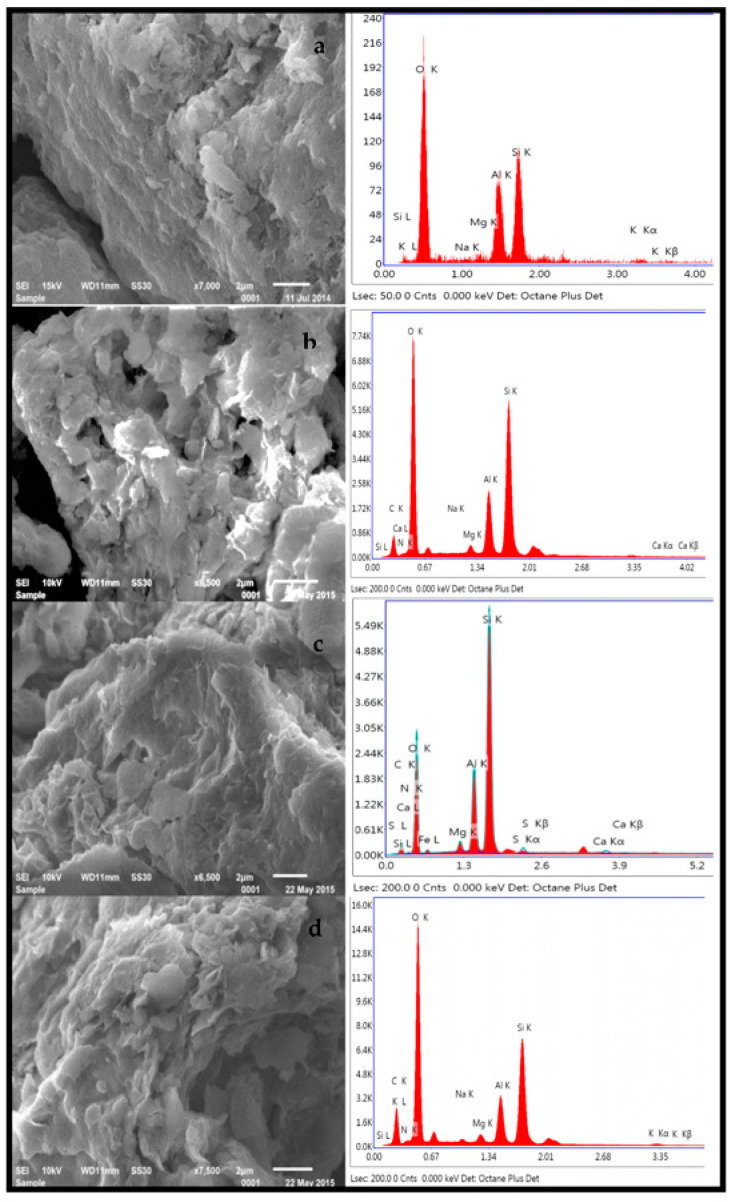
SEM images of the (**a**) pristine Mt, (**b**) CPC–Mt, (**c**) Mt–SO dye, and (**d**) CPC–Mt–SO dye.

Figure 8 presents the SEM images and energy-dispersive X-ray analysis of the pristine and organo Mt before and after the adsorption of dye. 

The pristine Mt had a nonporous surface (Figure 8a), but after modification with ammonium ions, the surface became porous and clay layers became more apart, which may have been due to the intercalation of CPC in the interlayers of Mt (Figure 8b). When the SO dye interacted with pristine Mt, a more compact structure of Mt was found (Figure 8c), whereas in the case of the organo Mt, the porosity disappeared to some extent, which may have been due to the adsorption of dye on the surface of the organo Mt (Figure 8d). The SEM EDX studies showed the peaks of the C and N elements in the organo Mt, which confirmed the surfactant’s presence in the clay interlayer. The percentage of C and N increased in the organo Mt–SO dye after interacting with the organo Mt, indicating the adsorption of the SO dye on the surface of the organo Mt.

## 4. Application of Clay-Based Nano Pigment for Colored Polymeric Membrane

As the Introduction section discusses, nano pigments are composite materials synthesized using clay and organic dyes. These nano pigments are used as reinforcing agents in polymeric materials to form polymer membranes. Due to the hydrophilic nature of Mt, they do not show good compatibility with a polymer matrix due to poor dispersion. Therefore, for better compatibility and a homogenous dispersion, the clay surface needs to be modified using a cationic surfactant [36,37,38]. 

**Figure 9:**
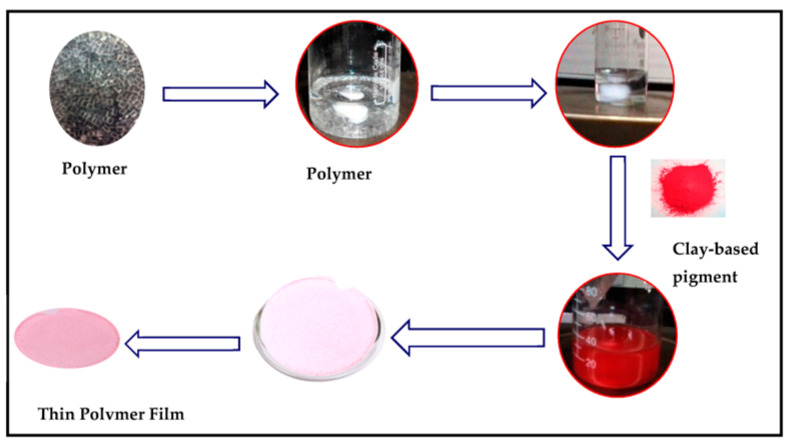
Synthesis of a polymeric membrane.

**Figure 10:**
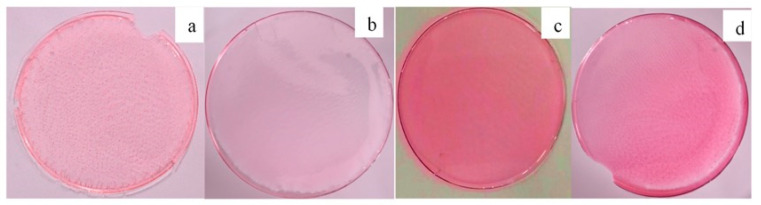
Picture of the (**a**) polymeric membrane containing Mt-based clay-based nano pigments, (**b**) polymeric membrane containing 1% OMt-based clay-based nano pigments, (**c**) 3% OMt-based clay-based nano pigments, and (**d**) 5% OMt-based clay-based nano pigments.

To fabricate a polymeric membrane, synthesized clay-based nano pigments were added to the polymeric solution under constant stirring. Initially, 5 g of PMMA polymer was dissolved in 25 mL of tetrahydrofuran solvent under continuous stirring (5–6 h) using magnetic stirring (300 rpm). After the complete dissolution of the polymer, 1%, 3%, and 5% of the clay-based nano pigments (relative weight of the polymer) were added into the polymeric solution and left to stir until the complete dispersion of the nano pigments was attained. The whole procedure was performed at a temperature of 25 °C. The solution was poured into Petri dishes and left at room temperature until complete evaporation of the solvent. The polymer films were taken out of the Petri dishes after complete evaporation of the solvent (Figure 9 and Figure 10). The membranes obtained were 3–5 μm thick with a 5 cm diameter.

The color intensity of the membrane is directly proportional to the dye loading onto the clay/organo clay and the amount of nano pigments dispersed in the polymeric solution. On increasing the amount of nano pigments in the polymer matrix, the transparent polymer film converts into a translucent polymer film with the visibility of the individual particles in the polymer films, which might be due to the agglomeration of nano pigment particles, resulting in their poor dispersion (Figure 10).

Hence, it is true that SO dye, being cationic in nature, shows more interaction with pristine Mt due to the electrostatic force of attraction between the positively charged dye and the negatively charged clay. Due to the hydrophilic nature of pristine clay, they did not disperse well in the polymer matrix, resulting in the visibility of the particles. Therefore, to ensure better compatibility and a homogenous dispersion with the polymer matrix, it is necessary to modify the surface of pristine clay minerals with a cationic surfactant. 

### 4.1. XRD Pattern of PMMA Polymeric Membrane and Clay Based Nano Pigments Containing Polymeric Membrane

**Figure 11:**
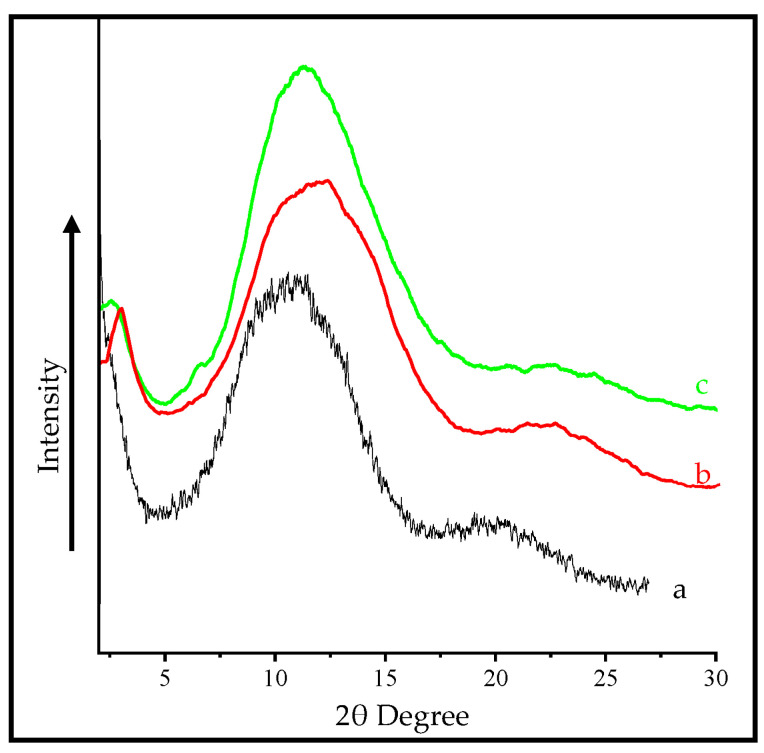
XRD pattern of the (a) pure PMMA polymeric membrane, (b) PMMA polymeric membrane containing Mt-based nano pigments, and (c) PMMA polymeric membrane containing OMt-based nano pigments.

Figure 11 shows the XRD pattern of the pure PMMA polymeric membrane and nano pigment-based PMMA polymeric membrane. The pure PMMA polymeric membrane showed the characteristic diffraction peak at a 2θ value of 15.43° with an interlayer spacing of 5.73 Å. After dispersing the nano pigments, the diffraction peak shifted toward the left, resulting in an increase in the basal spacing. In the case of the polymeric membrane containing Mt- and OMt-based SO nano pigments, the diffraction peak was found at a 2θ value of 3.90° and 2.84°, indicating an increase in the interlayer spacing at 22.64 Å and 31.37 Å, which was due to the presence of the PMMA polymer between the interlayer spacing of Mt and OMt as a single layer chain. The obtained interlayer spacing of the PMMA polymeric membrane containing the clay-based nano pigment was due to the penetration of the polymer between the clay platelets. The intercalation of the PMMA polymer into the interlayer of nano clay was further confirmed by Kumar et al. [39]. 

### 4.2. FTIR Spectra of PMMA Polymeric Membrane and Clay-Based Nano Pigments Containing Polymeric Membrane

**Figure 12:**
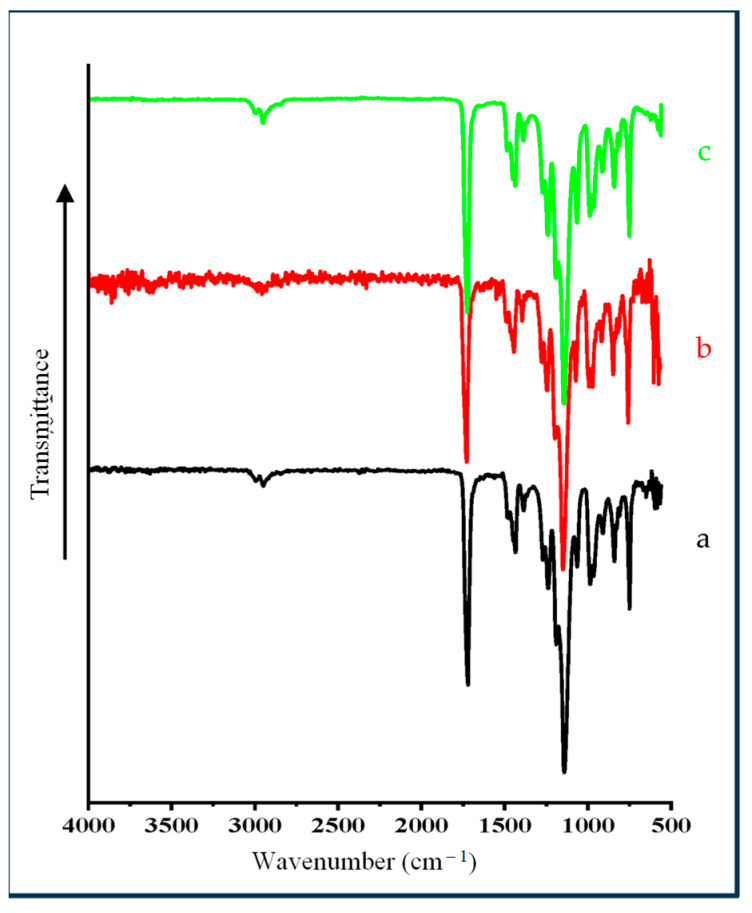
FTIR spectra of the (a) pure PMMA polymeric membrane, (b) PMMA polymeric membrane containing Mt-based nano pigments, and (c) PMMA polymeric membrane containing OMt-based nano pigments.

Figure 12 shows the FTIR spectrum of polymeric membrane and nano pigments based PMMA polymeric membrane It is evident that all of the FTIR spectra of the PMMA polymeric film and membrane and nano pigment-based PMMA polymeric membrane were almost similar, due to the presence of PMMA in all polymeric membranes. The vibration bands at 3000 and 2923 cm^−1^ corresponded to C–H stretching of the methyl group while the characteristics bands at 1425 and 1347 cm^−1^ were due to the C–H symmetric and asymmetric stretching modes. The bands at 1700 cm^−1^ and 1135 cm^−1^ were due to the carbonyl group of ester present in PMMA polymer. The vibration bands at 564, 805, and 955 cm^−1^ were due to Si–O–Si bending and Si–O–Si symmetric stretching. In the case of the PMMA polymeric membrane containing clay-based nano pigments, vibration bands were found in the same regions as the PMMA polymeric membrane. The vibration band around 805 cm^−1^ (Si–O–Si symmetric stretching) was found close to the band at 820 cm^−1^, due to the C–C stretching of PMMA.

### 4.3. High-Resolution Transmission Electron Microscopy (HR-TEM)

**Figure 13:**
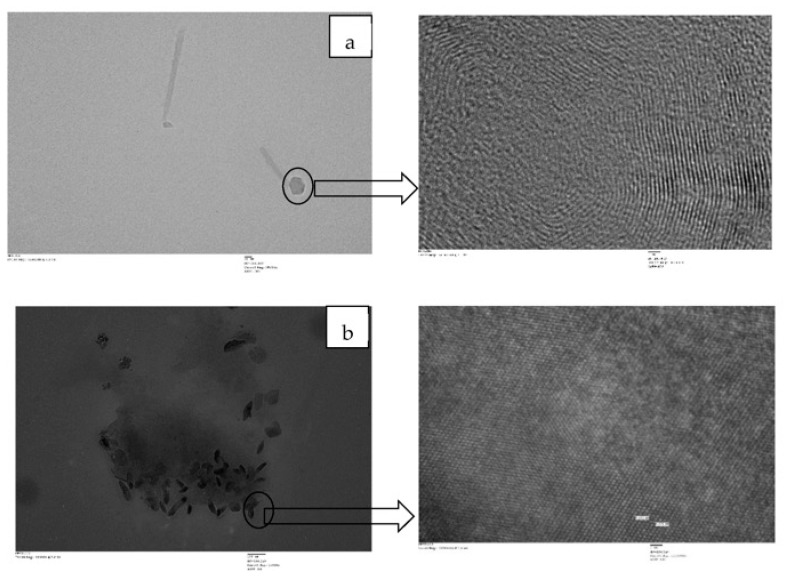
TEM images of the (**a**) PMMA polymeric membrane containing Mt-based nano pigments and the (**b**) PMMA polymeric membrane containing OMt-based nano pigments.

TEM analysis was undertaken to delineate the dispersion of clay-based nano pigments into the polymet matrix. The dark lines indicate the stacked clay platelets and the rest of the area represents the organic matrix (Figure 13). The hydrophilic nature of clay minerals and the presence of the chain length of the organic modified in the clay mainly decide the extent of intercalation and exfoliation. Both images confirmed the intercalated structure of the PMMA polymeric membrane containing Mt- and OMt-based nano pigments.

### 4.4. Mechanical Properties of PMMA Polymeric Membrane

Table 1 shows the effect of pristine Mt and organo Mt-based SO nano pigments on the PMMA polymeric membrane. The reinforcement of the polymer matrix generally increased after the addition of clay, resulting in an improvement in the mechanical properties of the polymer matrix. It was observed from the table that the PMMA polymeric membrane containing nano pigments displayed enhanced tensile strength and Young’s modulus. In comparison with the pure PMMA polymeric membrane, the tensile strength increased by a factor of 4.2 and 6.4 whereas the Young’s modulus increased by a factor of 3.2 and 4.5 after the addition of Mt- and OMt-based nano pigments, respectively. The enhancement was due to the reduction in the brittleness in the polymeric membrane after the addition of nano pigments. In the case of the polymeric membrane containing OMt-based nano pigments, the tensile strength and Young’s modulus increased more than the polymeric membrane containing Mt-based nano pigments as organo clay is compatible with PMMA. The presence of organo clay also provides a strong interfacial shear stress, resulting in greater enhancement of the mechanical properties [40]. The increment in the mechanical properties of polyurethane on the dispersion of nano clay was also confirmed by Pandey et al. [41]. The mechanical properties of the PMMA polymer were enhanced with only 1% loading of a Co–Al layered double hydroxide [42]. 

## 5. Conclusions

A novel methodology was successfully developed to synthesize clay-based nano pigments using the batch extraction method. For the separation of SO dye from aqueous media, pristine Mt (108 mg g^−1^) was found to be more effective for the removal of Safranin O dye when compared to OMt (58 mg g^−1^) due to its negatively charged surface. Clay-based nano pigments were successfully synthesized at optimized conditions obtained during the batch extraction studies. XRD and FTIR studies confirmed the SO dye’s successful intercalation and surface interaction with Mt and OMt. The thermal stability of dye was also increased after interaction with clay/organo clay. Clay-based nano pigments were found to have better thermal stability and brightness of color when compared to pure dye. They can be successfully dispersed as reinforcing fillers in the polymer matrix to form a beautifully colored, transparent polymeric membrane. The XRD pattern confirmed the successful intercalation of the PMMA polymer into the interlayer of Mt/OMt, resulting in an increase in the interlayer spacing of Mt and OMt, which was further confirmed by the TEM images. Clay-based nano pigments have several advantages over organic dyes and there are still some issues with inorganic pigments, so these nano pigments are used as colorants for polymer materials to form a beautifully red-colored, transparent, thin polymer film with enhanced physico-chemical properties. Polymeric membranes have several potential applications like removing pollutants from wastewater, coating industries, and many more. The challenges related to light stability, thermal stability, environmental safety, and degradation of the polymeric membrane need to be addressed and compared with the membranes that are already present on the market.

## Figures and Tables

**Table 1:** Mechanical properties of the polymeric membranes.

S. No.	Sample Code	Stress (Pascal)	Strain (mm/mm)	Tensile Strength (MPa)	Young’s Modulus (MPa)
01	PMMA + SO	10.72	0.038	3.06	346.28
02	PMMA + MtSO	18.45	0.040	5.27	523.12
03	PMMA + OMtSO	27.61	0.045	7.89	748.25

## Data Availability

Data available on request.

## References

[1] Mahmoodi A., Ebrahimi M., Khosravi A., Eivaz Mohammadloo H. (2017). A hybrid dye-clay nano-pigment: Synthesis, characterization and application in organic coatings. Dyes Pigments.

[2] Tianyong Z., Xuening F., Jian S., Chunlong Z. (1999). Properties of copper phthalocyanine microencapsulated in polystyrene by phase separation. Dyes Pigments.

[3] Herbst W., Hunger K. (1997). Miscellaneous pigments. Industrial Organic Pigments, Production, Properties, Applications.

[4] Farha A.H., Al Naim A.F., Mansour S.A. (2023). Cost-Effective and Efficient Cool Nano pigments Based on Oleic-Acid-Surface-Modified ZnO Nanostructured. Materials.

[5] Micó-Vicent B., Ramos M., Luzi F., Dominici F., Viqueira V., Torre L., Jiménez A., Puglia D., Garrigós M.C. (2020). Effect of Chlorophyll Hybrid Nano pigments from Broccoli Waste on Thermomechanical and Colour Behaviour of Polyester-Based Bionanocomposites. Polymers.

[6] Ngamwonglumlert L., Devahastin S., Chiewchan N. (2017). Natural colorants: Pigment stability and extraction yield enhancement via utilization of appropriate pretreatment and extraction methods. Crit. Rev. Food Sci. Nutr..

[7] Zhang R., Zhou L., Li J., Oliveira H., Yang N., Jin W., Zhu Z., Li S., He J. (2020). Microencapsulation of anthocyanins extracted from grape skin by emulsification/internal gelation followed by spray/freeze-drying techniques: Characterization, stability and bioaccessibility. LWT.

[8] Serbanescu O.S., Pandele A.M., Miculescu F., Voicu S.I. (2020). Synthesis and characterization of cellulose acetate membranes with self-indicating properties by changing the membrane surface color for separation of Gd(III). Coatings.

[9] Bello O.S., Olusegun O.A., Njoku V.O. (2013). Fly Ash: An alternative to powdered activated carbon for the removal of eosin dye from aqueous solutions. Bull. Chem. Soc. Ethiop..

[10] Edraki M., Zaarei D. (2018). Evaluation of the anti-corrosion effect of clay-based nano pigments modified with organic azole compounds. Adv. Mat. New Coat..

[11] Mahmoodi A., Ebrahimi M., Khosravi A. (2016). Preparation a Blue Color Epoxy/Clay Nanocomposite with a Better Color Performance, Iran. https://civilica.com/doc/578462/certificate/print/.

[12] Fischer H.R., Batenburg L.F. (2003). Coloring Pigment.

[13] Marchante V., Benavente V., Marcilla A., Martínez-Verdú F.M., Beltrán M.I. (2013). Ethylene vinyl acetate/nanoclay-based pigment composites: Morphology, rheology, and mechanical, thermal, and colorimetric properties. J. Appl. Polym. Sci..

[14] Raha S., Ivanov I., Quazi N.H., Bhattacharya S.N. (2009). Photo-stability of rhodamine-B/montmorillonite nano pigments in polypropylene matrix. Appl. Clay Sci..

[15] Smitha V.S., Manjumol K.A., Ghosh S., Brahmakumar M., Pavithran C., Perumal P. (2011). Rhodamine 6G intercalated montmorillonite nano pigments–polyethylene composites: Facile synthesis and ultraviolet stability study. J. Am. Ceram. Soc..

[16] Trigueiro P., Pereira F.A., Guillermin D., Rigaud B., Balme S., Janot J.M., dos Santos I.M.G., Fonseca M.G., Walter P., Jaber M. (2018). When anthraquinone dyes meet pillared montmorillonite: Stability or fading upon exposure to light?. Dyes Pigments.

[17] Szadkowski B., Kuśmierek M., Kozanecki M., Nowakowska J., Rogowski J., Maniukiewicz W., Marzec A. (2023). Ecological hybrid pigments with improved thermal, light, and chemical stability based on purpurin dye and different minerals for applications in polymer materials. Dyes Pigments.

[18] Cavalcanti G.R., Rodrigues F., Zhuang G., Balme S., Janot J.M., Fonseca M.G., Jaber M. (2023). Inorganic-organic hybrid pigments based on carminic acid and clay minerals. Dyes Pigments.

[19] Hussain A.F., Halboos M.H. (2020). Adsorption of safranin dye from their aqueous solutions by using CA and Nano FeO/CA. J. Phys. Conf. Ser..

[20] Sayhood A.A., Mohammed H.J. (2015). Synthesis of new azo reagent for determination of Pd(II), Ag(I) and applied to enhance the properties of silver nano particles. Int. J. Chem. Sci..

[21] Ghosh I., Kar S., Chatterjee T., Bar T., Das S.K. (2021). Adsorptive removal of Safranin-O dye from aqueous medium using coconut coir and its acid-treated forms: Adsorption study, scale-up design, MPR and GA-ANN modeling. Sustain. Chem. Pharm..

[22] Azimvand J., Didehban K., Mirshokraie S. (2021). Safranin-O removal from aqueous solutions using lignin nanoparticle-g-polyacrylic acid adsorbent: Synthesis, properties, and application. Adsorpt. Sci. Technol..

[23] Shah K., Parmar A. (2018). Removal of Safranin O dye from synthetic wastewater by Activated Carbon prepared from Tamarind seeds. Int. J. Appl. Eng. Res..

[24] Didehban K.H., Mirshokraie S.A., Azimvand J. (2018). Safranin-O dye removal from aqueous solution using super-absorbent lignin nanoparticle/polyacrylic acid hydrogel. Bulg. Chem. Commun..

[25] Pakdel P.M., Peighambardoust S.J., Arsalani N., Aghdasinia H. (2022). Safranin-O cationic dye removal from wastewater using carboxymethyl cellulose-grafted-poly(acrylic acid-co-itaconic acid) nanocomposite hydrogel. Environ. Res..

[26] Sieren B., Baker J., Wang X., Rozzoni S.J., Carlson K., McBain A., Kerstan D., Allen L., Li L.Z. (2020). Sorptive Removal of color dye safranin o by fibrous clay minerals and zeolites. Adv. Mat. Sci. Eng..

[27] Shi Y., Wang X., Wang X., Carlson K., Li Z. (2021). Removal of Toluidine blue and Safranin O from single and binary solutions using Zeolite. Crystals.

[28] Chanra J., Budianto E., Soegijono B. (2019). Surface modification of montmorillonite by the use of organic cations via conventional ion exchange method. IOP Conf. Ser. Mater. Sci. Eng..

[29] Guegan T. (2018). Organoclay applications and limits in the environment. Comptes Rendus Chim..

[30] Kumari J., Singh A. (2016). Green synthesis of nanostructured silver particles and their catalytic application in dye degradation. J. Gen. Eng. Biotechnol..

[31] Avila M.C., Lick L.D., Comelli N.A., Ruiz M.L. (2021). Adsorption of an anionic dye from aqueous solution on a treated clay. Groundw. Sustain. Dev..

[32] Romdhane D.F., Satlaoui Y., Nasraoui R., Charef A., Azouzi R. (2020). Adsorption, modeling, thermodynamic, and kinetic studies of methyl red removal from textile-polluted water using natural and purified organic matter rich clays as low-cost adsorbent. J. Chem..

[33] Sivalingam R., Sivasamy A., Muthukaruppan A., Chandrasekar F. (2011). Synthesis and characterization of poly(n-vinyl-2-pyrrolidone)-organo-modified montmorillonite (OMMT) clay hybrid nanocomposites. J. Comp. Mater..

[34] Kumari N., Mohan C., Negi A. (2023). An investigative study on the structural, thermal and mechanical properties of clay-based PVC polymer composite films. Polymers.

[35] Elhossein A., Moawed A., Abulkibash B. (2016). Selective separation of Light green and Safranin O from aqueous solution using *Salvadora persica* (Miswak) powder as a new biosorbent. J. Saudi Chem. Soc..

[36] Franco-Urquiza E.A. (2021). Clay-based polymer nanocomposites: Essential work of fracture. Polymers.

[37] Yadav S., Yusoh K. (2016). Modification of pristine nanoclay and its application in wood-plastic composite. e-Polymers.

[38] Kumar M., Arun S., Upadhyaya P. (2015). Properties of PMMA/clay nanocomposites prepared using various compatibilizers. Int. J. Mech. Mater. Eng..

[39] Naderi-Samani H., Razavi R.S., Loghman-Estarki M.R., Ramazani M. (2017). The effects of organoclay on the morphology and mechanical properties of PAI/clay nanocomposites coatings prepared by the ultrasonication assisted process. Ultrason. Sonochem..

[40] Pandey S., Jana K.K., Aswal V.K., Rana D., Maiti P. (2017). Effect of nanoparticle on the mechanical and gas barrier properties of thermoplastic polyurethane. Appl. Clay Sci..

[41] Adak B., Butola B.S., Joshi M. (2018). Effect of organoclay-type and clay-polyurethane interaction chemistry for tuning the morphology, gas barrier and mechanical properties of clay/polyurethane nanocomposites. Appl. Clay Sci..

[42] Kumar M., Chakraborty S., Upadhyaya P., Pugazhenthi G. (2017). Morphological, mechanical, and thermal features of PMMA nanocomposites containing two-dimensional Co–Al layered double hydroxide. J. Appl. Polym. Sci..

